# Deficient supplies of drugs for life threatening diseases in an African community

**DOI:** 10.1186/1472-6963-7-86

**Published:** 2007-06-15

**Authors:** Norman N Lufesi, Marit Andrew, Ivar Aursnes

**Affiliations:** 1 Department of General Practice and Community Medicine (Section for International Health), University of Oslo, Oslo, Norway; 2 Alliance apotek (Alliance UniChem Norway AS), Oslo, Norway; 3 Department of Pharmacotherapeutics, University of Oslo, Oslo, Norway

## Abstract

**Background:**

In Malawi essential drugs are provided free of charge to patients at all public health facilities in order to ensure equitable access to health care. The country thereby spends about 30% of the national health budget on drugs. In order to investigate the level of drug shortages and eventually find the reasons for the drugs shortages in Malawi, we studied the management of the drug supplies for common and life threatening diseases such as pneumonia and malaria in a random selection of health centres.

**Methods:**

In July and August 2005 we visited eight out of a total of 37 health centres chosen at random in the Lilongwe District, Malawi. We recorded the logistics of eight essential and widely used drugs which according to the treatment guidelines should be available at all health centres. Five drugs are used regularly to treat pneumonia and three others to treat acute malaria. Out-of-stock situations in the course of one year were recorded retrospectively. We compared the quantity of each drug recorded on the Stock Cards with the actual stock of the drug on the shelves at the time of audit. We reviewed 8,968 Patient Records containing information on type and amount of drugs prescribed during one month.

**Results:**

On average, drugs for treating pneumonia were out of stock for six months during one year of observation (median value 167 days); anti-malarial drugs were lacking for periods ranging from 42 to138 days. The cross-sectional audit was even more negative, but here too the situation was more positive for anti-malarial drugs. The main reason for the shortage of drugs was insufficient deliveries from the Regional Medical Store. Benzyl penicillin was in shortest supply (4% received). The median value for non-availability was 240 days in the course of a year. The supply was better for anti-malarial drugs, except for quinine injections (9 %). Only 66 % of Stock Card records of quantities received were reflected in Patient Records showing quantities dispensed.

**Conclusion:**

We conclude that for the eight index drugs the levels of supply are unacceptable. The main reason for the observed shortage of drugs at the health centres was insufficient deliveries from the Regional Medical Store. A difference between the information recorded on the Stock Cards at the health centres and that recorded in the Patient Records may have contributed to the overall poor drug supply situation. In order to ensure equitable access to life saving drugs, logistics in general should be put in order before specific disease management programmes are initiated.

## Background

The effectiveness of drug supply systems in achieving a reliable supply of essential drugs needs to be continually and objectively assessed. The drugs management cycle involves four basic functions: selection, procurement, distribution and use [1]. At the centre of the cycle is a core management support system, and the entire cycle rests on the policy and the legal framework that establishes and supports the public commitment to ensure that essential drugs are accessible to the intended population. Malawi, which is mainly dependent on imported drugs, adopted in1987 the principle of an essential drug list for the public health sector, now containing about 384 drugs [2]. Since then the government has intended to provide drugs free of charge at all public health facilities. It spends $7 annually per person on health, of which about $2 (29%) is spent on drugs [3]. It is crucial that essential drugs actually reach patients in need of them. However, there is evidence that drug shortage is a major barrier to access to essential drugs in the sub-Saharan Africa [4-6]. Many studies addressing the barriers of access to essential drugs in developing countries have been poorly designed and did only focus on the prescribers' and users' perspectives and not on the management of drugs supply systems [7]. In this study, we investigated the management aspect of the drug supplies for common and life threatening diseases such as pneumonia and malaria in Lilongwe district. We verified that the numerous complaints and local newspaper articles were truly reflecting the shortage of drugs used to combat life-threatening diseases in the country. The state-controlled supply system requires each health centre to report the stock situation to the District Pharmacy each month. The health centres do not have a pharmacist or pharmacy technician on the staff. The District Pharmacy, which also does not have trained pharmacy staff, compares the reported stock situation with a stock size guideline and sends an order to the Regional Medical Store. After a certain time lag, the health centre receives the drugs. The aim of our study was to compare the real situation in the field with the ideal situation as described in the Malawi health policy and in global recommendations.

## Methods

### Study design

The study design was cross sectional and retrospective. One of the authors (NNL) visited a random selection of health centres, recorded the quantities of the selected drugs on the shelves and retrospectively summed up the ordering history for the same drugs throughout the course of one year at each centre. He also compared the stock situation with the Patient Records for one month and balanced the amount of drugs dispensed with the amount received.

### Study setting and selection of the health centres

There are 27 districts in Malawi. Bearing in mind the limited finances available for the study, we concentrated our investigations to the area of the capital city and its surroundings, i.e. Lilongwe, which is situated in the centre of the country. According to the population census of 1998, Lilongwe district has a population of 1,337,777 with an annual growth rate of 2.9%. About 67% of the population live in the rural and peri-urban areas and comprises a range of mixed races and tribes in the city and the peri-urban areas. The rural areas are mainly inhabited by the Chewa tribe who financially is dependent on seasonal small scale farming of tobacco while maize is their main source of food. The main religions in the district are Christianity and Islam with Islam being practised mainly in the city [8]. The Lilongwe District has one Central Hospital, 37 government health centres, about eight Christian Hospital Association of Malawi (CHAM) health facilities and some private clinics. The private clinics are mainly located in the city. Using the Research Randomizer [9] we selected several groups of governmental health centres with eight in each group and chose one of these groups for further study.

### Selection of the index drugs

We studied the logistics of eight essential and widely used drugs, which according to the treatment guidelines should be available in all health centres at all times. Of these drugs, five are used regularly to treat pneumonia and the three others to treat acute malaria. Both these diseases are common and life-threatening conditions that contribute substantially to local morbidity and mortality. The drugs are listed in Table 1 and the units in which they are ordered are specified. Paraldehyde is used for the management of convulsions that sometimes occur in patients with severe malaria, especially in children.

**Table 1:** The total amount of the eight drugs that were supplied to the eight health centres according to the 38 reports that were reviewed

	***Ordering units***	***Number of units ordered***	***Fraction received***
***Drugs for pneumonia***			
Amoxicillin tab	1000 × 250 mg	784	31 %
Benzyl penicillin injection	One 5 mL unit vial	1525	4 %
Cotrimoxazole tab	1000 × (400 mg + 80 mg)	1018	50 %
Chloramphenicol injection	1 g sodium succinate vial	483	15 %
Erythromycin tab	1000 × 250 mg	425	21 %
***Drugs for malaria***			
Pyrimethamine/sulfadoxine tab	1000 × (25 mg + 500 mg)	325	63 %
Paraldehyde injection	One 10 mL ampoule	2167	48 %
Quinine injection	One 600 mg ampoule	2100	9 %

### Data collection

Data were collected during a personal visit by NNL, accompanied by a technical assistant, to each of the eight health centres, after a letter of recommendation had been sent in advance from the District Health Office. The health centre employees were very cooperative when interviewed about the ordering system and provided access to Stock Cards, Drug Reports and Patient Records (see below) which are maintained manually. We conducted nine interviews (eight interviews with the persons in charges of medicines at the health centres and one interview with the pharmacist in charge) using a structured questionnaire that contained questions on the procurement process, inventory management, supervision, training and drug management policy. The data obtained from the Stock Cards, Drug Reports and Patient Records were manually entered into standardised forms, which had been pre-tested at a health centre other than one of the eight and adjusted as necessary. Subsequently the data were transferred to an Excel Worksheet (Microsoft).

#### The drug ordering and dispensing system

The health centres initiate the drug-ordering process by compiling monthly Drug Reports that are submitted to the District Pharmacy. These reports contain information on the quantities used of each drug during the previous month and the balance in hand. The District Pharmacy technician assesses the health centre's requirements, completes the order part of the Drug Report in accordance with given criteria and forwards it to the Regional Medical Stores. Malawi has three Regional Medical Stores, based in each of the three regions in the country. Each Regional Medical Store serves the government and CHAM health facilities in the respective region. The Regional Medical Store then supplies the ordered drugs directly to the health centre. When drugs are received at the health centre, the quantity received and date of delivery are entered on the respective Stock Cards and on the centre's own copy of the Drug Report. Emergency supplies can also be ordered, in which case the same procedure should be followed as when ordering normal supplies. When drugs are issued from the health centre store to the dispensary the date of issue and quantity issued are recorded on the Stock Card. Ideally, when patients receive drugs from the health centre dispensary the quantity of each drug dispensed and the date should be recorded in the Patient Records.

#### Our review of the Drugs Reports, Stock Cards and Patient Records

We reviewed the Stock Cards for each of the eight selected drugs in order to determine the availability over a period of one year (calculated as 360 days). The quantities of each drug specified on the Stock Card were compared with the actual stock on the shelves. A total of 58 Stock Cards were reviewed at the eight health centres. Six cards were missing. The accumulated observation time in days was 20,880 days (360 days × 58 Stock Cards). For each drug the number of normal supplies and of emergency supplies and the number and duration of out-of-stock situations were recorded.

In order to assess the level of service provided by the Regional Medical Store we reviewed 54 Drug Reports stating, both by the health centre and the Regional Medical Store, the quantities requested and the quantities received. Thirty eight reports were found to be eligible for determining the ability of the Regional Medical Stores to supply the requested drugs to the health centres. In addition, we reviewed 8 968 Patient Records containing information on the type and quantities of drugs prescribed and issued topatients during the month of December 2004.

### Data analysis

All calculations regarding the number of days that the drugs were out of stock, the number of normal and emergency supplies received by the health centres, and drugs delivered to patients according to the Patient Records were carried out in Microsoft Excel. The total dosage of all the drugs presented in tablet or capsule forms was calculated as the product of the dose, frequency of administration and duration of treatment. For the injectable drugs, one vial or ampoule was regarded as the total dosage because according to the records the patients had received the drug in question only once. The number of days a drug was out of stock was calculated by counting the days from the date the drug was recorded on the Stock Card as being out of stock to the date the drug was recorded as being in stock again. We used the SPSS for descriptive analysis and for comparing the distribution of the different variables among the health centres and among the drugs. We used the non-parametric Kruskal-Wallis test because the distribution of the data was found to be non-normal. The differences were considered to be statistically significant if the P-value was < 0.05.

### Permissions and ethical clearance

The study was approved by the National Health Sciences Research Committee of the Ministry of Health in Malawi. At District level, the District Health Officer gave us permission to proceed with the study. At the health centre-level, the person in charge gave us permission to collect the data. The data were kept confidential and all the health centres studied were kept anonymous.

## Results

**Figure 1:**
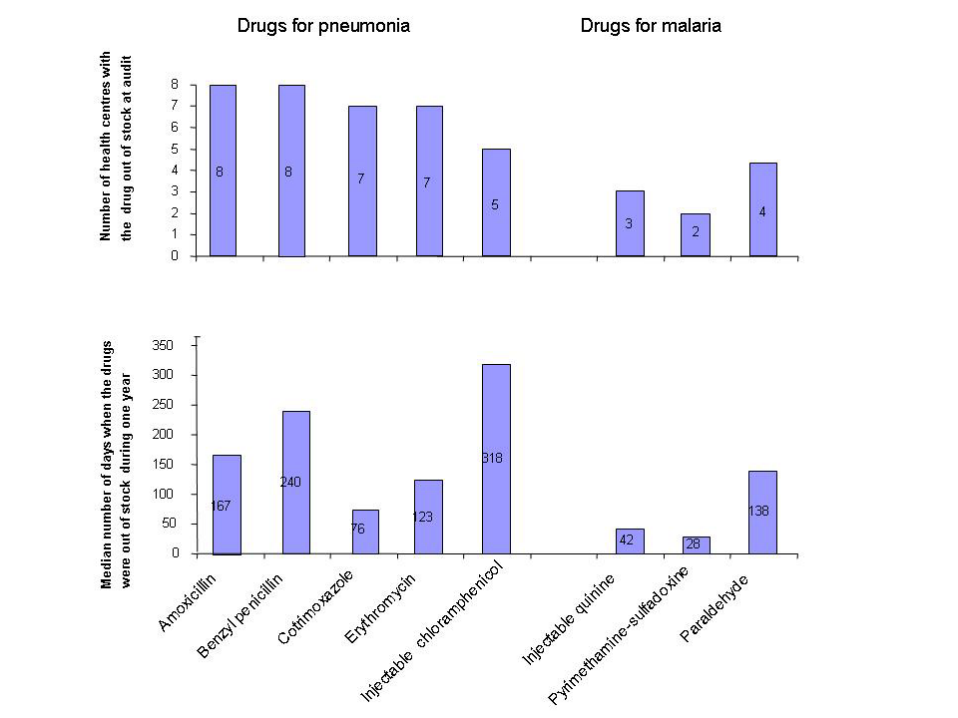
Deficiencies in drug supplies at 8 Malawian health centres.

It can be seen from Figure 1 that supplies of the selected drugs to the health centres were highly inadequate, the shortage being most pronounced for the drugs used to treat pneumonia. On average, these drugs were out of stock for six months during the one year of observation (median value 167 days). The situation was slightly better but still serious for anti-malarial drugs. On average these drugs were out of stock for less than one third of the year of observation (range 42 to 138 days). Figure 1 shows similarities between the cross-sectional data (i.e. numbers of health centres without the respective drugs on the shelves at the time of audit) and the retrospective findings (i.e. median numbers of days the respective drugs which according to the store records had been out of stock during the whole year of observation). Overall, the audit results gave a more worrying picture than did the retrospective data (see Figure), but here again the situation was somewhat better for the anti-malarial drugs than for the drugs used to treat pneumonia.

### Insufficient deliveries from the Regional Medical Store

It was found that the main reason for the observed shortage of drugs at health centre level was insufficient deliveries from the Regional Medical Store (see Table 1 and first row in Table 2).

**Table 2:** Probable reasons for insufficient drug supplies with comments, ordered according to assumed degree of importance

***Reason***	***Comment***
Insufficient deliveries from the Regional Medical Store	The median fraction of the ordered drugs received by the health centres was 18 % (see Table 1)
Stocked supplies not recorded as having been given to patients	For December 2004 we observed a median difference of 66 % between Stock Cards and Patient Records. The highest was for chloramphenicol (92 %) and lowest for benzyl penicillin (29 %)
Uneven distribution of drugs among health centres	Injectable drugs (quinine and chloramphenicol) were unequally distributed among health centres both at audit and throughout the year (see text)
Problems with certain therapeutic indications	Drugs for acute malaria were in better supply than drugs for pneumonia (see Figure)
Lack of training	None of the health workers managing the drugs in the eight health centres were trained in drug management nor in bookkeeping

Of the drugs used to treat pneumonia the supply level was lowest for benzyl penicillin, with only 4% of the requested quantity being received (Table 1). This corresponds with the observation that benzyl penicillin was not in stock at any health centre at the time of audit, and for all the health centres collectively the median value for being out of stock was 240 days out of one year (see Figure).

Anti-malarial drugs were in better supply, except for quinine injections where only 9 % of the ordered quantity was received (Table 1). This figure does not correspond with the seemingly better stock situation at the time of audit, when quinine injections were available at five of the eight health centres and where the median value for non-availability was 42 days in the course of one year (Figure).

### Stocked supplies not recorded as having been given to patients

From the 8968 Patient Records, we found that pyrimethamine/sulfadoxine (first line treatment of malaria) was the most commonly prescribed drug (60.4%) followed by cotrimoxazole (first line treatment of pneumonia) that was prescribed in 24.8% of all the cases. Chloramphenicol and paraldehyde injectables were the least prescribed drugs (0.1%). We found discrepancies between the utilisation of drugs on the Stock Cards and the Patient Records. The highest discrepancy was for chloramphenicol injectable (92%). The median discrepancy percentage for all drugs was 66%. The second row in Table 2 illustrates the discrepancy between the quantity of the drugs issued from the health centre stores to the dispensary and the quantity of the drugs delivered to patients, as stated in the Patient Records.

### Uneven distribution of drugs among health centres

Uneven distribution of drugs among the health centres may also have contributed to scarcity in some places. According to the records, the number of days a drug was out of stock varied from 0 to 122 days for injectable quinine and from 0 to 360 days for injectable chloramphenicol at the various health centres. Overall there was a statistically significant difference among the various health centres (P = 0.02, Kruskal-Wallis test). Similarly, the same drugs were found to be in stock at time of audit in quantities ranging from zero ordering units for injectable chloramphenicol at five health centres to 48 units at one health centre, and from zero ordering units of injectable quinine at three health centres to a maximum of 210 units at another health centre.

### Time taken from ordering to receiving of drugs and lack of training and supervision

The time taken between ordering and receiving the drugs, lack of appropriate skills for health workers managing drugs and lack of supervision may also have contributed to the shortage of drugs in the health centres. From the drug reports, we found that drugs took 8 to 105 days from the time of ordering to the time the drugs were actually received by the health centre. The median delay between ordering and supply was 37 days. Seven health centre persons in charge of medicines reported that drugs were usually received more than 28 days after the day the drugs were requested. From the interviews, we found that none of the health workers managing drugs were trained in basic drug management skills and that they were rarely supervised by the district pharmacist.

## Discussion

Although we expected the situation to be less than optimal, it was a surprise to find that the index drugs were on average unavailable to patients at health centres for six months out of a year in the case of pneumonia drugs and for three months out of a year in the case of anti-malarial drugs. Findings on the drugs available at time of audit agreed to a large extent with the stock situation throughout a full year. To our knowledge, no similar investigation has been carried out in Malawi before and we do not know of comparable data from other sub-Saharan countries with similar distribution systems [6]. In our opinion, since the selected health centres were a random sample from the health centres in one specific region (Central Region) and were served by one Regional Medical Store, our findings could at the least be representative of the situation in the region as a whole. Drug shortages have been reported to be a major problem throughout Malawi [10,11], and as there is a national drug management system, our findings at large may well apply to the whole country.

The main limitation of the present study is that it was restricted primarily to observations and interviews at the health centre level. We noted that in four out of five instances no drugs were supplied in response to a normal order. So far we can only speculate on the reasons for this situation. A point of interest is that many acute situations may have been solved by sending an emergency order to the District Pharmacy. This is not an ideal situation, however, since the health centre concerned then receives drugs intended primarily for other users. We also question the guidelines stating that the quantity of a drug ordered should be based on the consumption during the previous month [12]. When there are no drugs at the health centre to consume, this is not a feasible procedure. With frequent out-of-stock situations the requirements should be based on demographic and morbidity data [13].

We found gross discrepancies between the Stock Cards filled in at the health centres and the Patient Records. The most likely explanation is that drugs are de facto dispensed to patients but not recorded as such, which constitutes lack of compliance with procedures. The procedures themselves may be out of touch with real life challenges in a busy outpatient clinic. In any case, this kind of "malpractice" makes it difficult to deal with allegations of theft which, according to newspaper reports [14,15] and studies in other countries [16,17] cannot be excluded to take place.

It is possible to envisage ways to improve the system: training the personnel in bookkeeping combined with supportive supervision, a type of intervention proven to be effective in Zimbabwe [18]; decentralization of the drug budget to the District, which might allow the Districts to buy drugs from other sources when the government medical store fails to supply, as was found to be the case in Uganda [4]; and a small co-payment from patients that can be used to replenish the supplies at health centre level. As the Bamako initiative has proved, this increases the availability of drugs [19].

One of our findings was an unequal distribution of the drugs in stock among the various health centres. This may indicate lacking focus on equitable access to essential drugs. The intended role of the district pharmacies is to enforce some sort of national, criteria based harmonization. One measure could be to strengthen the district pharmacies authority and ability to respond to such information and to perform some kind of reallocation of drugs when needed.

We noted that the supply of anti-malarial drugs was somewhat better than that of drugs used to treat pneumonia. There could be various explanations for this, including the fact that malaria, along with AIDS and tuberculosis, has received special governmental and international attention through the Global Fund for HIV/AIDS, Tuberculosis and Malaria and the Roll Back Malaria Global Partnership. No comparable initiatives exist for pneumonia, despite this being a major killer of children [20]. We know several initiatives have been taken to provide drugs for treating AIDS in Malawi [21]. A well-functioning drug supply system is a critical factor for success in any disease management program. AIDS initiatives in Malawi may well profit by or even depend on a joint effort in reviewing and improving the drug supply infrastructure in the country.

## Conclusion

We conclude that for the index drugs, anti-malarial drugs and drugs used to treat pneumonia, the levels of supply are unacceptable. To a large extent these drugs were out of stock during our visits to the various health centres and had also been out of stock for extensive periods during the previous year. Also the distribution level among the health centres showed large variations, resulting in both lack and inequity of access for patients.

The main reason for the observed shortage of drugs at health centre level was insufficient deliveries from the Regional Medical Store. We have not explored the reasons for these shortcomings. On the one hand the public sector may be procuring insufficient drugs to meet the country's needs, on the other hand there may be a leakage of drugs from the public sector into the private sector, such as has been described in other countries [5,22].

We also found a consistent and large difference between Stock Card recordings at the health centres and Patient Records. This practice makes it difficult to exclude that leakage or theft and may have contributed to the overall drug supply situation. Another contributory factor may have been the observed lack of training in core principles of drug management as well as basic record keeping. In order to ensure equitable access to life saving drug, logistics in general should be put in order before specific disease management programmes requiring a reliable drug supply chain are initiated.

## Competing interests

The author(s) declare that they have no competing interests.

## Authors' contributions

The reported data originate from a thesis for the Master Degree in International Health presented by NNL to the Faculty of Medicine, University of Oslo, in June 2006. Details from the prescription analysis can be found in the thesis book. Based on an original idea by NNL, all three authors planned the study and examined the data after they had been collected by NNL. IA drafted the manuscript and developed it with the help of the other two authors. All three authors have read and approved of the final manuscript.

## Pre-publication history

The pre-publication history for this paper can be accessed here:


